# Gains through selection for grain yield in a winter wheat breeding program

**DOI:** 10.1371/journal.pone.0221603

**Published:** 2020-04-28

**Authors:** Dennis N. Lozada, Brian P. Ward, Arron H. Carter

**Affiliations:** 1 Crop and Soil Sciences Department, Washington State University, Pullman, WA, United States of America; 2 USDA-ARS Plant Science Research Unit, Raleigh, NC, United States of America; Institute of Genetics and Developmental Biology Chinese Academy of Sciences, CHINA

## Abstract

Increased genetic gain for complex traits in plant breeding programs can be achieved through different selection strategies. The objective of this study was to compare potential gains for grain yield in a winter wheat breeding program through estimating response to selection *R* values across several selection approaches including phenotypic (PS), marker-based (MS), genomic (GS), and a combination of PS and GS (PS+GS). Ten populations of Washington State University (WSU) winter wheat breeding lines including a diversity panel and F5 and double haploid lines evaluated from 2015 to 2019 growing seasons for grain yield in Lind and Pullman, WA, USA were used in the study. Selection was conducted by selecting the top 20% of lines based on observed yield (PS strategy), genomic estimated breeding values (GS), presence of yield “enhancing” alleles of the most significant single nucleotide polymorphism (SNP) markers identified from genome-wide association mapping (MS), and high observed yield and estimated breeding values (PS+GS). Overall, PS compared to other individual selection strategies (MS and GS) showed the highest mean response (*R =* 0.61) within the same environment. When combined with GS, a 23% improvement in *R* for yield was observed, indicating that gains could be improved by complementing traditional PS with GS within the same environment. Validating selection strategies in different environments resulted in low to negative *R* values indicating the effects of genotype-by-environment interactions for grain yield. MS was not successful in terms of *R* relative to the other selection approaches; using this strategy resulted in a significant (*P* < 0.05) decrease in response to selection compared with the other approaches. An integrated PS+GS approach could result in optimal genetic gain within the same environment, whereas a PS strategy might be a viable option for grain yield validated in different environments. Altogether, we demonstrated that gains through increased response to selection for yield could be achieved in the WSU winter wheat breeding program by implementing different selection strategies either exclusively or in combination.

## Introduction

The challenge to develop higher yielding, climate resilient, disease- and pest-resistant, and more nutritious crops has never been more urgent considering anticipated continuing global population growth over the next 30 years [1]. As such, improving genetic gain or performance for important traits such as yield, disease resistance, and adaptation in staple crops such as wheat (*Triticum aestivum* L.) has been the goal of many breeding programs. Genetic gain is the predicted change in mean value of a trait within a population under selection [2] and is represented by the “breeder’s equation”, ΔG = (σ_a_)(i)(r)/L, where ΔG is the change in genetic gain; σ_a_ is the additive genetic variation within the population, *i* is the selection intensity, *r* is the selection accuracy, and *L* is the number of cycles per year [3]. To increase genetic gain, an increase in the phenotypic variance, accuracy of selection, and selection intensity, or a decrease in generation time for cultivar development, is necessary [4]. Phenotypic, genomic, and marker-based selection approaches could be used to increase either of the factors mentioned to achieve improved gains.

In bread wheat, phenotypic selection for superior genotypes, characterized primarily by a “non-shattering” phenotype, began during its domestication [5]. This “unconscious” breeding resulted from the unintentional selection of lines that were more adapted and productive under early farming practices [6]. “Empirical” and “scientific” breeding followed the “unconscious”, which resulted in the development of wheat lines with improved characteristics in breeding programs [6]. Currently, plant breeders have access to advanced genome and phenotypic-based selection strategies to fast-track genetic improvement and increase gains for key traits in wheat [1].

Several studies have evaluated the gains which could be achieved by applying different selection strategies particularly for increasing resistance to specific diseases in wheat. Rutkoski et al. [7] compared gains for phenotypic and genomic selection for quantitative stem rust resistance and observed that genomic selection could perform as well as phenotypic selection for stem rust resistance improvement but can result in less genetic variance within a population over time. Significant gains using marker-assisted selection for Fusarium head blight (FHB) resistance were also observed in the University of Minnesota wheat breeding program due to the introgression of a major quantitative trait locus for FHB resistance (*Fhb1*). Using closely linked and diagnostic markers for *Fhb1* caused a 27% reduction in disease symptoms throughout the breeding program [8]. In another study, FHB severity in winter wheat was reduced by 6 and 5% using phenotypic and marker-aided selection, respectively [9]; whereas marker-assisted breeding for reduced disease severity and grain deoxynivalenol (DON) content resulted in higher gains in FHB resistance on an annual basis in spring wheat [10]. Both studies observed a large variation for FHB resistance in the marker-selected lines demonstrating the need to complement marker-based selection with phenotypic selection to further enhance gains.

Grain yield is a complex trait controlled mainly by many loci with small effects [11–13] making yield more difficult to select for than disease resistance. Improvement in grain yield, however, remains the prime emphasis of many wheat breeding programs [14], and therefore it is necessary to measure gains achieved through different breeding and selection strategies. As there are now several selection approaches available to plant breeders, we were interested in quantifying the possible gains for grain yield which could be attained when these methods are implemented either alone or in combination with others in a winter wheat breeding program. The objective of this study was to compare the projected gains for yield resulting from using different selection strategies in the Washington State University (WSU) winter wheat breeding program. Empirical datasets for grain yield collected from over 2,200 WSU winter wheat breeding lines grown from 2015 to 2019 were evaluated. The different selection strategies assessed included phenotypic, marker-based, genomic, and a combination of phenotypic and genomic selection. Potential gains for yield represented as the response to selection *R* were calculated for these selection strategies. Validation for grain yield response *R* was also conducted using different environments for the different selection approaches.

## Materials and methods

### Winter wheat populations

A total of ten winter wheat breeding populations adapted to the US Pacific Northwest were used in the study. These populations included an association mapping panel (AMP), two F5 biparental populations, two double haploid (DH) biparental populations, and five populations of winter wheat lines evaluated for preliminary yield trials (Prel) as validation populations for yield. The AMP consisted of 456 lines evaluated in Lind (LND) and Pullman (PUL) WA, USA between 2015 and 2018. Significant soil crusting delayed the growth of the winter wheat lines in LND in 2016 and hence the AMP was not evaluated for this site-year. The F5 populations were comprised of 61 and 501 lines planted in 2017 in LND (LND17_F5) and PUL (PUL17_F5), WA respectively. The DH populations were evaluated in LND and PUL in 2018 and consisted of 447 (LND18_DH) and 759 (PUL18_DH) winter wheat breeding lines. The Prel validation populations comprised of lines selected and advanced from the 2017 and 2018 F5 and DH populations and were evaluated in LND and PUL, WA.

### Phenotypic data collection and analyses

Grain yield (in t ha ^-1^) was assessed by harvesting whole plots using a Zurn^®^ 150 combine (Waldenburg, Germany). Adjusted yields were calculated using an Augmented Complete Block Design (ACBD) with replicated checks and un-replicated test genotypes in each block using a custom R script [15]. Field plots were 2.5 m in length, with each entry covering ~3.7 m^2^ area and ~260 plants per m^2^ where each block contained ~37 and 45 plots. The winter wheat line ‘Eltan’ [16] was used as a check in LND and ‘Madsen’ [17] was used as a check in PUL for the 2015–2018 growing seasons for the AMP. Checks for the LND17_F5 included the lines ‘Bruehl’ [18], Eltan, ‘Otto’ [19], ‘Jasper’ [20], Madsen, and ‘Xerpha’[21], whereas ‘Brundage’[22], Jasper, Madsen, ‘Puma’[23], ‘UI Bruneau’, and ‘Xerpha’ were used for the PUL17_F5 population. Jasper, Otto, and Xerpha were used as checks for LND18_DH; whereas Jasper, Madsen, Puma, and Xerpha were used as checks for the PUL18_DH panel.

Adjusted values for yield were calculated employing two statistical models following Lozada and Carter [24]. Briefly, the models used were:

$$Y_{ij}=\mu +B_{i}+G+C+I+\varepsilon_{ij}$$ (1)

$$Y_{ijkl}=\mu +G+C+I+E_{i}+I\;x\;E_{i}+G\;x\;E_{i}+C\;x\;E_{i}+B_{k}(E_{i})+\varepsilon_{ijkl}$$ (2)

where Y is the trait of interest; μ is the effect of the mean; B_i_ is the effect of the *i*^th^ block; G corresponds to the un-replicated genotypes; C is the effect of the replicated checks on each block; E_i_ is the effect of the *i*^th^ environment; I is the effect of the identifier of the checks; this was used to differentiate the effects of one check over the other checks, as well as the number of checks present on each block; I x E_i_, G x Ei, and C x E_i_ are the effects of check identifier by environment, genotype by environment, and check by environment interactions, respectively; B_k_(E_i_) is the effect of block nested within each environment; and ε is the standard normal error [15]. Best linear unbiased estimates (BLUEs) were calculated for individual environments (Eq (1)), whereas best linear unbiased predictors (BLUPs) were computed for the combined analyses across locations (Eq (2)). Factors were considered fixed when calculating BLUEs whereas effects were regarded as random for calculating BLUPs.

### Genome-wide association study and genomic predictions

SNP genotyping was conducted using genotyping-by-sequencing (GBS) using the protocol of Poland et al. [25,26] through the NC State University Genomics Sciences Laboratory in Raleigh, NC, USA. The restriction enzymes *MspI* and *PstI* were used for GBS. Short-read sequences were aligned to the Chinese Spring IWGSC RefSeq v1.0 [27] using the Burrow-Wheeler Aligner (BWA) 0.7.17 [28] followed by SNP calling using TASSEL-GBS v. 5.2.43 [29,30]. SNP markers were filtered for minor allele frequency (MAF) of > 0.05 and 10% missing data and were designated with their chromosome location and base pair position, e.g. “S1A_497083519”. Imputation of missing data was done using the linkage disequilibrium *k*- nearest joining imputation (LD-kNNi) function [31] in TASSEL v.5.2.25. After filtering and quality control, 16,233 markers (genotype data 1, GD1; S1 File), where 15,853 (97.7%) of which aligned to contigs which were mapped to chromosomes, remained and were used for genome-wide association study (GWAS) using a fixed and random effects circulating probability unification (FarmCPU; [32]) kinship (*K*) only with no PC included in the model in R [33]. The optimal number of PCs to be included in the GWAS model was determined using the “model selection” function in GAPIT [34], which uses a Bayesian information criterion to select which is the optimal model to use. Based on analyses, a PC = 0 was the optimal number across the datasets (S1 Table), and therefore a *K* only model was used for GWAS. Minimal deviations from the quantile-quantile (QQ) plots were also observed, indicating that the GWAS model used already sufficiently accounted for familiar relatedness and population structure, and hence PC was excluded in the model (S1 Fig). SNP loci were declared to be significant under a Benjamini-Hochberg false discovery rate (FDR) [35] threshold of 0.05 to control false positive results. The percent phenotypic variation explained (R^2^) by each significant SNP locus was calculated using a stepwise regression model in JMP^®^ Genomics v.8.1 [36], where the R^2^ value when a marker was removed from the regression model was subtracted from the total R^2^ when all the significant SNP markers were fitted in the model.

Genomic predictions and genomic estimated breeding value (GEBV) calculations were implemented in the iPAT (Intelligent Prediction and Association Tool) package [37], where a ridge regression best linear unbiased prediction (RRBLUP) selection model [38] was trained using the AMP to predict the yield performance of WSU F5 and DH winter wheat breeding lines for independent validations. This prediction model shrinks marker effects towards zero with a common variance [38]. RRBLUP uses the ‘mixed.solve’ function in the form: **y** = **Xβ** + **Zu** + ε, **u** ~ N (0, **K**σ^2^_*u*_), where **X** is a full-rank design matrix for the fixed effects, **β**; **Z** is the design matrix for the random effects **u**, **K** is a semidefinite covariance matrix, obtained from markers using the ‘A.mat’ (additive relationship matrix function); residuals are normal with a mean of zero and constant variance; and **u** and ε independent [38].

A total of 11,089 high-quality GBS-derived SNP markers common to both the AMP and the validation sets (genotype data 2, GD2; S2 File) were used for genomic predictions. GD2 was a subset of GD1 which was used to perform association analyses using the AMP. Phenotypic data for yield in the validation populations (F5 and DH breeding lines) were masked by representing them as “NA” during each analysis. Two GS scenarios were implemented, namely, a standard GS (GS1) considering all markers as contributing equally to the polygenic background effect, and a GWAS-assisted GS scheme (GS2) using the AMP as training population. Predictive ability for the independent validations were calculated as the Pearson correlation between GEBV and adjusted yield for the F5 and DH wheat breeding lines. For the GWAS-assisted GS (GS2), the top five most significant SNP markers based on an FDR of 0.05 were fitted in an RRBLUP genomic prediction model as fixed effects in iPAT. A total of seven BLUE and two BLUP yield datasets were used for GWAS and genomic predictions. Relatedness between the diversity training panel and winter wheat test lines were assessed using Rogers genetic distances calculated in JMP Genomics v.8.0.

### Correlation between GEBV for one year and observed yield in the succeeding year

The relationships between calculated breeding values from one year and corresponding adjusted yield values in the succeeding year were evaluated by calculating GEBV of the lines in the AMP and comparing them to their adjusted yield in the next growing season (e.g. GEBV for PUL2015 was compared to adjusted yield in PUL2016), which in essence, is also test of genotype-by-environment interactions. GEBVs were calculated by performing a five-fold cross-validation for the AMP, where 80% of the lines were used to predict the remaining 20% using an RRBLUP model in iPAT for the GS1 scenario. The Pearson correlation coefficients between GEBV and adjusted yield were calculated.

### Selection strategies and response to selection

Different selection approaches for grain yield, namely phenotypic (PS), marker-based (MS), genomic (GS), and phenotypic + genomic (PS+GS) selection were compared in this study. For PS, the top 20% of the F5 and DH lines based on adjusted values for yield were selected. In MS, lines having five yield “enhancing” loci identified from association mapping using the AMP were selected. These loci represented the five most significant SNP markers based on a Benjamini-Hochberg FDR of 0.05 across datasets. In the GS approach, the top 20% of the breeding lines having the highest GEBV were identified through independent predictions by training the AMP to predict yield of the F5 and DH breeding lines (GS1). In another GS scenario, five of the most significant markers identified from association mapping using the AMP were included in the selection model as fixed effects to predict yield for the breeding lines using an RRBLUP model (GS2). Finally, for the PS+GS approach, lines having the top 20% highest adjusted grain yield and the highest GEBV were selected for both GS1 (PS+GS1) and GS2 (PS+GS2). The average of the adjusted yield of the corresponding lines selected for each of the selection strategy was reported. Comparisons between mean yield achieved by applying the different selection approaches were also compared to the mean of the check lines.

Gains achieved through each selection approach were represented as the response to selection, *R*, calculated as *R* = *H*^*2*^S [39], where *H*^*2*^ is the broad-sense heritability calculated as $H^{2}=\frac{\sigma_{g}^{2}}{\sigma_{g}^{2}+\sigma_{e}^{2}}$, where $\sigma_{g}^{2}$ and $\sigma_{e}^{2}$ are the variances due to genotype and error, respectively; and *S* is the selection differential, calculated as *S* = μ_Selected_-μ_Unselected_, where μ_Selected_ is the mean yield for the lines with a selection strategy implemented and μ_Unselected_ is the mean yield of the lines without selection applied [40]. Both values for broad and narrow-sense heritability can be used to predict *R* [41]; however to capture the maximum variation due to genetic effects arising from using different populations of wheat breeding lines from the breeding program, *H*^*2*^ values were used to calculate response *R*. Significance tests using *t*-test were conducted to compare the mean response *R* of the different selection strategies for yield.

### Validation of selection response for grain yield in different environments

Different selection strategies, namely, PS, GS, and PS+GS were validated in other environments by comparing the mean yield achieved by the top 20% of lines selected from the test populations (LND17_F5, LND18_DH, PUL17_F5, and PUL18_DH) through the observed phenotypic values (PS), genomic breeding values (GS), and a combination of observed and genomic values (PS+GS) with their performance in the next growing seasons. To assess the performance of lines selected in each population in different environment, five different validation populations of winter wheat breeding lines evaluated in preliminary yield trials (Prel) from the WSU winter wheat breeding program were used, including LND18_F5_Prel, LND19_DH_Prel, PUL18_F5_ Prel, PUL18_F5_Prel 2, and PUL19_DH_Prel.

## Results

### Distribution of markers and significant marker-trait associations

SNP markers used were distributed in all chromosomes of wheat, with genome B having the largest number of markers (7,201; 44.4%), followed by genome A (6,244; 38.5%), and D (2,408; 14.8%) (S2 Table). Chromosomes 2B, 5B, and 7A had the greatest number of markers (1,306, 1,223, and 1,179 SNP markers, respectively), whereas 4D (92), 6D (284), and 1D (304) had the least among the chromosomes. There were 380 SNP markers (2.3%) that aligned to contigs which were not mapped to any chromosome. A total of 24 significant marker-trait associations (MTAs) distributed across 14 chromosomes were identified for yield in the AMP under a kinship model and an FDR of 0.05 (Table 1). FDR adjusted *P*-values for the significant markers ranged between 6.43E-06 (*S1A_535858090*) and 0.048 (*S3B_482345832*), whereas allele effects ranged between -0.39 and 0.26. The significant MTAs had an average minor allele frequency of 0.32. No SNP locus was identified to be significant across all locations; nevertheless, five loci were identified as having the most significant *p*-value and being significant across most of the locations (Table 1). These five SNP markers, located on chromosomes 1A, 3B, 6B, and 7A, were subsequently used to validate the MS approach.

**Table 1:** SNP markers associated with grain yield identified in a diverse training panel of US Pacific Northwest winter wheat lines (*N* = 456 lines).

SNP	Dataset	*P-*value	FDR adj. *P*-value, *q* ^a^	Minor allele frequency	Percent variation explained, R^2^	Reference
**S1A_497083519**	PUL15	1.91E-06	0.01	0.38	0.02	
**S1A_535858090** ^**b**^	**PUL18**	**4.13E-10**	**6.43E-06**	**0.34**	**0.03**	
**S1B_8150831**	PUL18	3.90E-06	0.01	0.14	0.05	[43]
**S2A_752287563**	LND17	7.92E-06	0.02	0.36	0.01	[43]
**S2B_239862383**	LND17	1.38E-06	0.01	0.37	1.0E-04	[43]
**S2B_775486161**	PUL18	5.71E-06	0.01	0.41	0.02	[43,44]
**S2D_639821303**	LND17	1.52E-05	0.03	0.18	0.02	
**S2D_642029978**	LND17	5.66E-06	0.02	0.08	7.0E-04	
**S3A_22831895**	LND18	4.68E-06	0.04	0.42	0.02	[42,43]
**S3A_567971108**	PUL15	3.04E-06	0.01	0.19	0.01	[42,43]
**S3B_482345832**	PUL18	2.51E-05	0.05	0.47	2.5E-03	[43]
**S3B_561570016**	**PUL18**	**4.49E-07**	**3.0E-03**	**0.26**	**0.02**	[43]
**S3B_818284683**	**PUL15**	**6.39E-07**	**5.0E-03**	**0.47**	**0.02**	[43]
**S3D_325690**	LND17	2.25E-06	0.01	0.21	3.3E-03	
**S5B_29125444**	LND17	4.18E-07	0.01	0.08	5.0E-04	[43]
**S5B_47592949**	PUL18	2.83E-06	0.01	0.31	0.01	[43]
**S5B_679577399**	LND18	8.40E-06	0.04	0.32	0.02	[43]
**S6A_601959488**	LND17	1.42E-05	0.04	0.21	1.2E-03	[43,44]
**S6B_118986455**	**LND18**	**1.05E-08**	**1.0E-04**	**0.15**	**0.03**	[44]
**S6B_33331876**	PUL18	4.43E-06	0.01	0.50	8.0E-04	[44]
**S7A_545581556**	PUL15	1.44E-05	0.03	0.46	0.06	[42–44]
**S7A_61774265**	**PUL15**	**6.86E-08**	**1.0E-03**	**0.36**	**0.02**	[42–44]
**S7B_711208053**	PUL15	1.29E-06	0.01	0.45	0.02	
**S7D_635365239**	PUL15	6.48E-06	0.02	0.46	0.01	[44]

^a^ FDR- False discovery rate

^b^ Significant SNP markers in bold text were included in the prediction model as fixed effects for a GWAS-assisted genomic selection scenario (GS2)

### Predictive ability and genomic estimated breeding values for grain yield

**Fig 1:**
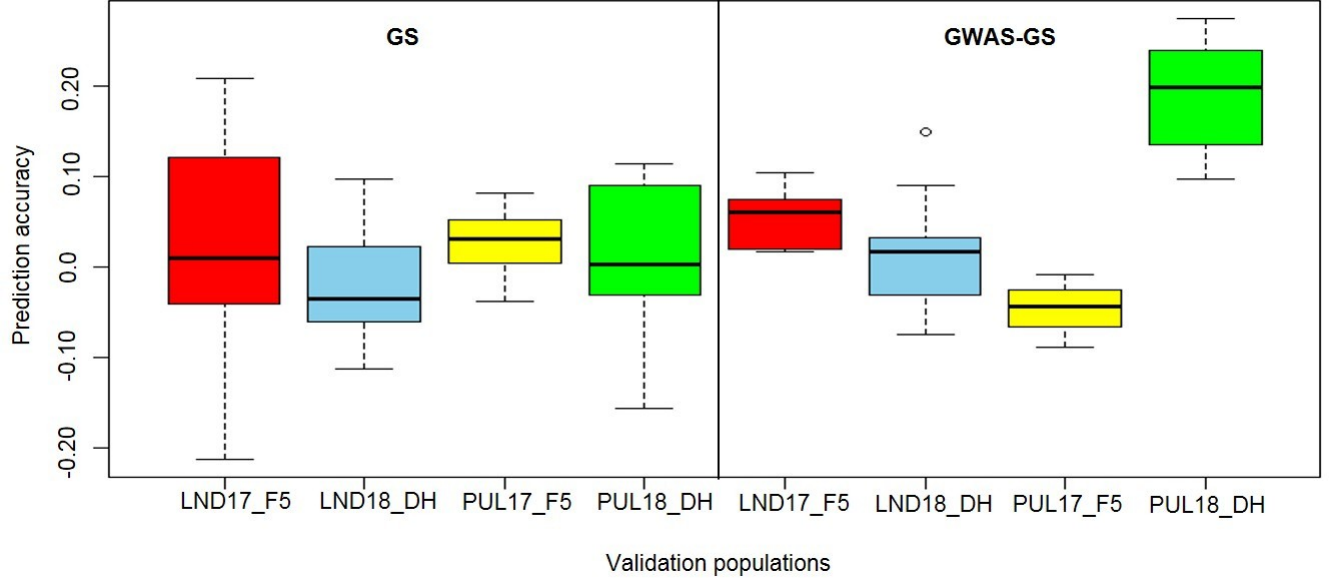
Box plots for prediction ability across a standard genomic selection approach using RRBLUP (GS1) and a GWAS-assisted GS scheme (GS2) for grain yield in a winter wheat breeding program using the AMP as training population.

Prediction ability for the GS1 scenario under independent validations were low, ranging from -0.21 (PUL16 predicting LND17_F5) to 0.21 (PUL15 predicting LND17_F5) across the wheat breeding lines (Fig 1). Overall, higher accuracies were observed for predicting the F5 lines compared with the DH populations (accuracy of 0.03 vs. 0.0002). No significant differences were observed for accuracies when models were trained using the LND and PUL datasets (0.01 vs. 0.02). Predicting LND17_F5 and LND18_DH wheat breeding lines using LND datasets resulted in a mean prediction ability of -0.01 whereas using PUL17_F5 and PUL18_DH as validation populations resulted in a mean predictive ability of 0.01 (S3 Table). Across environment predictions using the LND yield datasets to predict PUL17_F5 and PUL18_DH populations resulted in a mean of 0.04, whereas using PUL datasets to predict LND17_F5 and LND_18 DH resulted in a mean of 0.02. BLUP datasets showed an advantage over BLUE datasets for predictions (0.02 vs. 0.01) across different validation populations. Mean grain yield GEBV for all the breeding lines across each dataset ranged between 2.22 (LND15 as training dataset) and 9.99 (PUL18 as training dataset) for GS1 (S4 Table).

Predicting grain yield using the five SNP markers identified from GWAS as fixed effects in the model (GS2) did not result in significant differences in mean accuracy overall, although it resulted in an increase in predictive ability (0.05 vs. 0.02). Significant differences (*P <* 0.05) for mean prediction ability, nonetheless, were observed for PUL17_F5 and PUL18_DH. Prediction ability for GS2 ranged between -0.09 (PUL18 predicting PUL17_F5) and 0.27 (PUL15 predicting PUL18_DH). Highest mean prediction ability across datasets was observed for PUL18_DH (0.19), followed by LND17_F5 (0.05), LND18_DH (0.02), and PUL17_F5 (-0.04). Predicting yield using BLUP datasets did not give advantage over to using BLUEs for predictions. In contrast to GS1, within environment predictions resulted in a 50% gain in mean prediction ability compared to predicting across environments. Similar with the GS1 scenario, the highest mean GEBV for yield was observed for PUL18 (7.68) whereas the lowest was observed for LND15 (1.74) (S5 Table).

**Fig 2:**
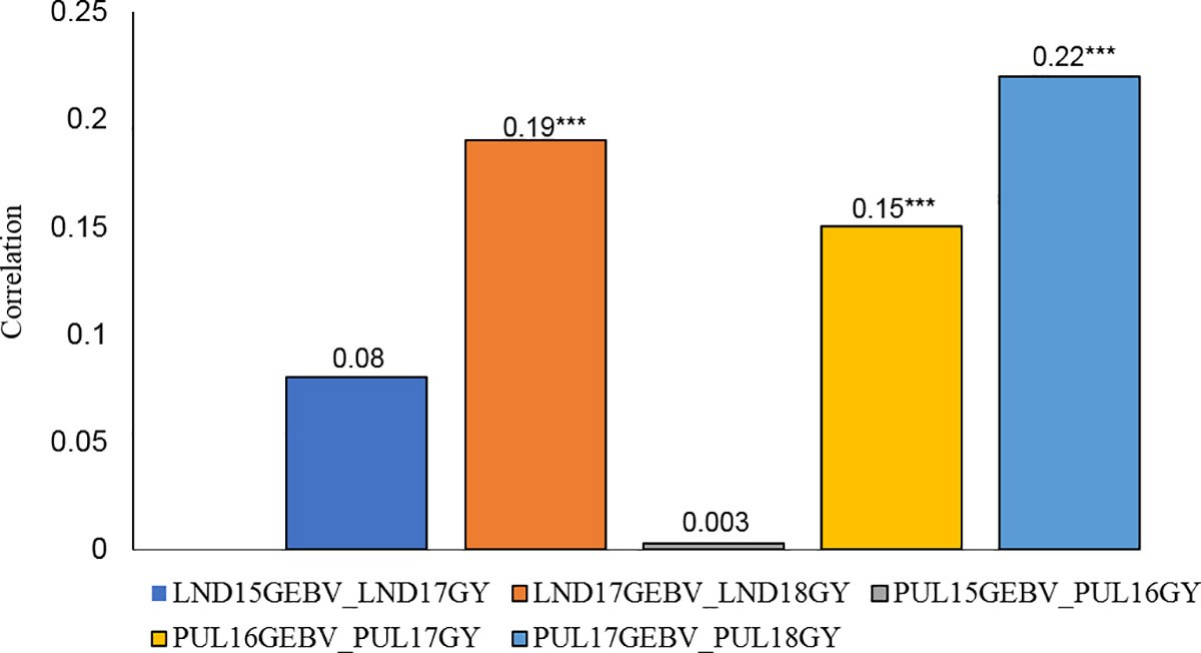
Correlation between genomic estimated breeding values (GEBV) and adjusted yield for consecutive growing seasons for a diverse association mapping population (AMP) of US Pacific Northwest winter wheat evaluated in Lind (LND) and Pullman (PUL), WA from 2015–2018. ***- Significant correlation at *P* < 0.0001.

Correlations between GEBV and adjusted yield for the winter wheat breeding lines were low to high, ranging between 0.08 (LND18) and 0.71 (PUL combined across years, PUL_Com). Likewise, significant associations (*P* < 0.0001) between GEBV and yield were observed across growing seasons for the diverse population of US Pacific Northwest winter wheat lines (AMP) (Fig 2). Correlation coefficients ranged from 0.003 (PUL15GEBV_PUL16GY) to 0.22 (PUL17GEBV_PUL18GY).

### Response to selection across different selection strategies

Selection of the top 20% of winter wheat lines was based on adjusted yield and GEBV for the F5 and DH wheat breeding populations in the same environment. This resulted in selecting 91 (AMP), 12 (LND17_F5), 90 (LND18_DH), 100 (PUL17_F5), and 150 lines (PUL18_DH) (Table 2). The highest average value for response to selection, *R*, was 0.63 for PS+GS1. Using paired *t*-test, the mean *R* of the PS+GS (PS+GS1 and PS+GS2) approaches were observed to be significantly (*P* < 0.0001) different with that of the means for GS1, GS2, and MS. The mean *R* for PS+GS, however, was not significantly different than that of PS, although there was a 5% increase in *R* for PS+GS1. Negative mean values for selection response were observed for both GS1 (-0.003) and MS (-0.35) (Tables 2 and 3). No line was selected under the LND17_F5 population using an MS approach, whereas there were four, 86, and 11 lines containing five favorable alleles for the most significant SNP markers identified from GWAS for LND18_DH, PUL17_F5, and PUL18_DH, respectively. Validating grain yield in different environments by comparing the yield of the selected 20% in each population evaluated in another environment (i.e. the following growing season) based on PS resulted in low to negative selection responses (Table 2), where *R* values ranged between -0.28 and 0.26. Using LND18_F5_Prel for validating LND17_F5 yield through a PS approach resulted in significant (*P* < 0.0001) differences in mean *R* compared to other populations under a paired *t*-test. Mean *R* for both GS and PS+GS for yield validated in different environments were also low, ranging between -0.29 and 0.08 (S3 Table).

**Table 2:** Response to selection *R* based on phenotypic selection (PS) for grain yield validated in different environments for US Pacific Northwest winter wheat.

Population	Validation pop.	Pop. mean (without selection)	Mean (with selection)	Selection differential ^a^	*H*^2^ ^b^	Response to Selection ^c^
**LND17_F5**	LND18_F5_Prel	3.58	5.33	1.75	0.15	0.26
**LND18_DH**	LND19_DH_Prel	4.57	4.06	-0.50	0.56	-0.28
**PUL17_F5**	PUL18_F5_Prel	8.66	8.80	0.14	0.13	0.02
**PUL17_F5**	PUL18_F5_Prel 2	8.66	8.72	0.06	0.13	0.01
**PUL18_DH**	PUL19_DH_Prel	9.62	9.27	-0.35	0.53	-0.19

^a^ Calculated as the difference between the mean yield of lines with selection and mean yield without selection, S = μ_Sel_-μ_Unselected_

^b^ Broad-sense heritability

^c^ Calculated as *R = H*^*2*^*S*

**Table 3:** Response to selection, *R* for GEBV-based selection (GS1 and GS2) strategies within the same environment for grain yield in US Pacific Northwest winter wheat. *R* values calculated based on the mean of population without selection applied.

Test population	*H*^*2*^	Training population (AMP) ^a^								
		LND15	LND17	LND18	LND_Com	PUL15	PUL16	PUL17	PUL18	PUL_Com
*GS1* ^*b*^										
**LND17_F5**	0.15	3.0E-03	-0.02	0.01	0.03	0.05	-0.07	-0.02	0.02	0.02
**LND18_DH**	0.56	0.08	-0.10	-0.03	-0.12	-0.02	0.0	0.13	-0.12	-0.12
**PUL17_F5**	0.13	-0.02	-9.0E-03	0.02	-0.01	0.02	0.02	0.01	-0.01	0.03
**PUL18_DH**	0.53	-0.13	0.11	0.11	0.02	0.27	0.04	-0.10	-0.19	-0.01
***GS2*** ^***c***^										
**LND17_F5**	0.15	0.003	-0.01	-0.03	-0.05	0.06	0.02	4.0E-04	0.09	0.08
**LND18_DH**	0.56	0.08	-0.10	-0.03	-0.13	-0.02	-2.5E-03	0.13	-0.12	-0.12
**PUL17_F5**	0.13	-0.02	-9.0E-03	0.02	-0.01	0.02	0.02	0.01	-4.0E-03	0.19
**PUL18_DH**	0.53	-0.13	0.11	0.06	0.02	0.27	1.10	1.09	1.09	-0.02

^a^ AMP-Association mapping panel

^b^ GS1- standard genomic selection

^c^ GS2- GWAS-assisted genomic selection

Using both PS+GS1 and PS+GS2 strategies, with mean *R* of 0.63 and 0.53 respectively, were more advantageous in terms of response than MS, GS1, and GS2 within the same environment (Table 4). Using GWAS-derived SNP markers as fixed effects in the prediction model in the GS2 scenario resulted in higher mean *R* (0.10) compared to GS1 (-0.003). The number of lines selected on both PS and GS ranged from 0 to 44 for both PS+GS1 and PS+GS2 approaches. There were no breeding lines selected for both PS and GS scenarios when PUL16 was used to predict LND17_F5. There were 16 values for *R* (44%) for the PS+GS1 that were greater than the *R* value using the PS alone. On the other hand, only 13 *R* values (36%) for the PS+GS2 were greater than the *R* for PS (Table 4, underscored and boldfaced values).

**Table 4:** Response to selection, *R*, for phenotypic + genomic (PS+GS1 and PS+GS2) selection strategies and number of lines selected in combining both approaches for selection (in parentheses) of yield within the same environment in US Pacific Northwest winter wheat. *R* values calculated based on the mean of population without selection applied.

**Test population**	***H*^*2*^**	**Training population (AMP) ^a^**								
***PS+GS1***		**LND15**	**LND17**	**LND18**	**LND_Com**	**PUL15**	**PUL16**	**PUL17**	**PUL18**	**PUL_Com**
**LND17_F5**	0.15	0.11 (1)	0.14 (1)	0.15 (2)	**0.17** (3)	**0.17** (2)	0	**0.18** (2)	0.16 (3)	0.15 (2)
**LND18_DH**	0.56	**1.06** ^b^ (24)	0.96 (19)	**1.05** (21)	0.97 (18)	**1.06** (18)	0.96 (13)	**1.00** (26)	**1.01** (16)	**1.01** (16)
**PUL17_F5**	0.13	0.17 (15)	0.17 (20)	0.18 (27)	0.19 (16)	**0.20** (29)	0.18 (26)	0.18 (29)	0.19 (19)	0.19 (31)
**PUL18_DH**	0.53	**1.14** (32)	1.09 (38)	**1.12** (30)	**1.12** (35)	**1.11** (44)	**1.10** (29)	1.07 (27)	1.05 (23)	**1.11** (28)
***PS+GS2***										
**LND17_F5**	0.15	0.11 (1)	0.14 (1)	-0.02 (2)	0 (3)	0.08 (2)	- (0)	**0.18** (2)	0.10 (3)	0.14 (2)
**LND18_DH**	0.56	**1.06** (24)	0.96 (19)	**1.05** (21)	0.97 (18)	**1.06** (18)	0.96 (13)	**1.00** (26)	**1.01** (16)	**1.01** (16)
**PUL17_F5**	0.13	0.17 (15)	0.17 (20)	0.18 (27)	0.19 (16)	**0.20** (29)	0.18 (26)	0.18 (29)	0.19 (19)	0.19 (31)
**PUL18_DH**	0.53	**1.14** (32)	1.09 (38)	**1.17** (39)	**1.12** (35)	**1.10** (44)	**1.10** (29)	-0.20 (30)	-0.37 (38)	1.09 (28)

^a^ AMP-Association mapping panel

^b^ Values in boldface and underlined indicate that the response is greater than that of response for PS within the same environment

Significant differences (*P* < 0.05) were observed between the mean *R* values for PS, GS, and MS when the mean of the checks was compared to the mean yield for the population under selection (S6–S8 Tables). Mean *R* values for PS and PS+GS1 both resulted in a 56% gain in response when compared to the mean of the checks. A total of 16 selection response values (44%) for the PS+GS1 showed higher *R* compared to the PS, whereas no *R* value for the PS+GS2 was observed to be greater than that for PS alone (S8 Table).

## Discussion

This study reports significant marker-trait associations for grain yield, and the potential gains, represented as the response to selection *R*, which could be achieved through employing different selection strategies for grain yield in a winter wheat breeding program. Among the selection strategies evaluated were phenotypic (PS), marker-based (MS), genomic (GS), and the combination of PS and GS (PS+GS) under independent predictions within the same environment and in different environments. The potential of integrating selection approaches to achieve increased gains for grain yield in winter wheat breeding programs was observed when evaluating within the same environment, whereas a PS approach might be advantageous for achieving optimal gains for yield when selecting across different environments.

### Significant marker-trait associations for grain yield

A GWAS approach identified 24 significant SNP markers associated with grain yield distributed across 14 chromosomes. Many of these coincided with previously identified MTAs in wheat [42–44], indicating the role of these genomic regions in controlling yield. A locus on chromosome 3A (S3A_22831895) was within ~1.7Mbp of SNP 3A_21102523, which was associated with yield in a population of spring wheat lines evaluated under high-temperature conditions [43]. The SNP markers on chromosomes 1A, 2D, 3D, and 7B identified in this study could be potentially novel loci controlling grain yield, as they have not previously been reported for wheat. All these associations were environment specific, and no SNP markers were associated across all environments or with the yield BLUP values. In augmented designs, BLUP trait values derived from combining trials from multiple locations may be limited by the estimation of variance components [45]. Only minor effect loci were detected across datasets, although five SNP markers were still identified as being the most significantly associated with grain yield and therefore used in subsequent MS approaches. Altogether, our GWAS results further support the complex genetic architecture for grain yield in wheat [11–13].

### Response to selection across different approaches

Response to selection *R* is one of the measures of genetic gain [46]. In the current study, phenotypic selection (PS) showed an advantage over MS and GS approaches in terms of *R*. Selecting a portion of lines (i.e. top 20%) based only on the adjusted yield for the F5 and DH wheat breeding lines showed a potential 24% gain on yield relative to the mean of the unselected population. It was nevertheless observed that combining PS with different GS approaches (PS+GS1 and PS+GS2) under independent predictions for some of the datasets resulted in improved *R* relative to that of the PS (Table 4). This indicates the possibility of achieving increased gains when selecting for lines having high observed yield and high estimated breeding values (GEBV) within the same environment. Our results were consistent with previous observations for increased *R* values when GS was combined with a PS approach in selecting for winter wheat lines with high grain yield and tolerance to snow mold, where a 10% and 7% gain in response where observed for the traits, respectively, compared to using PS alone [47,48]. Therefore, when performing selections within the same environment for traits with lower heritability, breeders could consider both information from PS and GS (through GEBV) to select lines. Selecting entries having high observed yield and high breeding values could give an opportunity to choose lines that are likely to do well across environments and years in comparison to lines selected based on phenotype alone in a single year [49]. One caveat for using the PS+GS approach for selection in low heritability traits, however, is that in some instances there would be no lines that have both high GEBV and high observed yield, as in the case of using the PUL16 dataset for predictions. This issue could be circumvented by evaluating more lines and increasing the selection intensity in the breeding program, which could improve the chances of selecting lines having both high phenotypic value and high GEBV.

Selection responses achieved by integrating GWAS-derived markers as fixed effects in the prediction model (GS2) were not significantly different than that of a standard GS approach (GS1) considering only a polygenic background effect, although 17% improvement in the mean *R* was observed. This demonstrated the potential to increase gains by incorporating fixed effect markers in the model, consistent with previous studies [50,51]. It should be noted that the markers used as fixed effects in the selection model were identified to be significant only in the training population (AMP) to disregard the effect of “inside trading,” which was previously observed to cause overestimated accuracies for FHB resistance in wheat [40]. These inflated accuracies under “inside trading” are attributed to the bias caused by using significant markers that are identified using data obtained from the validation set of lines for which predictions are being generated [40]. Using simulations, Bernardo [52] previously showed that incorporating markers with *R*^*2*^ greater than 10% in the model should give an advantage in increasing the accuracy. In the present study, significant loci with *R*^*2*^ greater than 10% were not identified. Nevertheless, we still observed a positive effect of including significant markers on the predictive ability for grain yield. In addition to using GWAS-derived markers for prediction, the inclusion of genetically correlated, highly heritable traits from high-throughput field phenotyping in the prediction model have been observed to improve selection accuracy for grain yield in wheat [53–56].

Negative responses were observed for marker selection (MS) for wheat breeding lines using independent SNP markers identified from association mapping using the AMP, indicating the inefficiency of using this approach exclusively for the selection of grain yield. Some reasons for this could be the low heritability of the trait, allelic variation, variable marker effects among populations, and linkage between the marker and trait of interest. Additionally, there were no LND17_F5 lines having favorable allele combinations for the most significant yield-related SNP loci, which further demonstrates the difficulty of performing selections based on an MS approach (Table 2; S9 Table). Nevertheless, its potential for selecting lines with high genetic potential particularly when combined with other approaches should not be discounted. Previously, some QTL validation studies for grain yield in wheat showed the potential of using allele specific assays such as KASP^®^ [57] to select for lines with high yield potential. Lozada et al. [58] developed marker assays for yield and component traits and used a diverse panel of spring wheat lines from the International Maize and Wheat Improvement Center (CIMMYT) in Mexico to validate the effects of yield-related loci previously identified in southern US winter wheat. They eventually showed the potential of developing molecular marker assays that could select for spring wheat lines with improved yield potential. In the context of genomic predictions for FHB related traits in wheat, the use of independent SNP markers (i.e. markers identified using a different mapping population) was previously observed to have neutral or reducing effects on selection accuracy [40].

Improvement for grain yield using MS approaches remains a challenge due to its genetic complexity, heritability, and the effects of genotype-by-environment interactions compared to disease resistance traits which are controlled by relatively few QTL with major effects [59]. In contrast, marker-assisted validation, marker-aided backcrossing, and marker-assisted gene pyramiding have been successfully implemented for different traits such as leaf rust resistance, powdery mildew resistance, and pre-harvest sprouting tolerance, among other traits [60]. In the present study, using MS alone did not result in improved gains, though the utility of MS in combination with GS and PS is an area for potential future study.

Validating grain yield of winter lines selected either through the PS, GS, or an integrated PS+GS approach in different environments altogether did not result in an increase for *R* across the datasets. This indicates the relevance of using similar environments when evaluating selections [24,53,61] as differences of QTL effects in environments used for predictions could affect prediction accuracy [61]. With the diversity of environments and years for the multilocation trials in the Pacific Northwest, differences in the effects of QTL for yield were expected and genotype-by-environment interactions were present strongly affecting *R*. While some positive responses were seen using one location to predict the other, in general the low heritability of grain yield and the high environmental variation between environments and across years makes this selection method difficult. In other production regions with less environmental variation, prediction across locations may be more feasible.

### Prediction accuracy and GEBV-based selection for grain yield

Predictive ability for grain yield under independent validations was also low. The genetic relatedness between the training and test populations was the main driver of predictive ability in the current study. Average Rogers’ genetic coefficient between the training and test populations was 0.31, indicating genetic differences among the populations used (S10 Table) which could have resulted in low prediction accuracy. These results were consistent with a related study [24], where low prediction ability was also observed for grain yield in winter wheat using the same set of winter wheat lines evaluated under partial least square regression prediction models. This demonstrates the complexity of using training panels for prediction within breeding programs. Although the AMP consisted of 456 lines from regional Pacific Northwest breeding programs (which are routinely used as parents in cross-hybridizations), and with over half of the lines coming from the WSU program, it was still limited in usefulness for genomic prediction due to low genetic relatedness with the F5 and DH validation panels that were being selected.

Using GEBV alone for selection was not successful relative to the PS and PS+GS approaches in terms of response to selection for within and across environment selections for grain yield. Negative *R* values were observed for almost 50% of the datasets for both GS1 and GS2. Relying exclusively on GEBV for performing selections should therefore be taken with care, as some lines predicted to have high GEBV could have low yield. Correlations between GEBV and observed yield between a year and the next growing season under cross-validations using the AMP were in general low, indicating that high GEBV sometimes do not translate to high observed phenotypic values. This is especially true when evaluating across years due to the possible effects of genotype-by-environment interactions, especially for low heritability traits. In the context of selecting new parental lines based on GEBV alone, it was recently observed that selecting for high FHB resistance in winter wheat was not reliable, as only 19% of lines (9 out of 47) predicted by GEBV belong to the best 10% for FHB resistance [62]. In another study, negative GEBV for yield were observed for synthetic hexaploid spring bread wheat lines evaluated across heat-stressed and irrigated environments [63]. Selection for drought tolerance in maize using GEBV, in contrast, has resulted in rapid genetic gain and positive selection responses through using molecular markers associated with high yield under drought stress [64].

While selecting lines based on GEBV alone should be considered with caution, the implementation of genomic selection in breeding programs should help increase the rate of genetic gain through a faster breeding cycle, higher selection intensity, and efficiency of genomic prediction approaches in integrating novel genetic material in wide-crosses and pre-breeding programs [65]. For GEBV to be more relevant in breeding programs, strategies that could help increase the selection accuracy, such as using genetically related populations, utilizing optimal training population composition and sizes, and employing ideal number of markers for predictions [66–69] should be implemented. Altogether, our results demonstrated that GEBV could still be used as a selection criterion for grain yield in winter wheat breeding, particularly for within environment selection for grain yield.

### Response to selection: Implications for breeding

Using different selection approaches in combination could result in increased response *R*, and hence genetic gain for complex traits. In the context of the WSU winter wheat breeding program, evaluating the selection approaches proposed here on breeding materials from earlier stages before the actual yield trials could also be performed to reduce costs associated with phenotyping and maximize gain achieved from genomic selection. Nevertheless, limitations were found when trying to select lines from one environment and predict their grain yield in another environment. Selection within the same environment was more successful. In earlier generations of selection, a PS+GS approach could result in better genetic gain for selections within the same environment. A PS strategy might still be more advantageous for selecting grain yield across different environments after the initial selection within a given environment.

## Conclusions

Gains in terms of response to selection *R* was compared for different selection strategies in a winter wheat breeding program. Phenotypic selection (PS) showed favorable responses to selection compared to genomic (GS) and marker-based selection (MS) approaches. Combining PS with GS showed a great potential in achieving higher *R* values compared to using either method alone for lines evaluated in the same environment. Validating selection responses in other environments, in contrast, resulted in negative *R*. We observed that GS, when combined with traditional PS for yield, could facilitate an increased response to selection within the same environment and ultimately could improve genetic gain in the WSU winter wheat breeding program. Breeders should therefore make important decisions based on the combination of one or more selection strategies to achieve optimal gains in plant breeding programs. For example, for complex traits evaluated in diverse environments, PS might be advantageous, whereas when evaluating within the same environment, an integrated PS+GS approach, where selections are based on both phenotypic and GEBV information, could result in optimal gains for grain yield. Careful consideration on which selection strategies to implement, depending on the traits being evaluated, cost, target environments, and available resources should altogether facilitate improved genetic gain for complex traits in winter wheat breeding programs.

## Supporting information

S1 Fig Quantile-quantile plots for grain yield. There were minimal deviations from the diagonal line (in red) indicating that the GWAS model (*K* only) was already able to capture population structure and genetic relatedness, and hence, principal components were excluded in the model. The SNP markers that deviate from the diagonal on the upper right-hand section of the graph are the loci significantly associated with the trait.
(TIF)

S1 Table Bayesian information criterion (BIC) values for different number of principal components (PC = 0 to PC = 3) across different yield datasets in the US Pacific Northwest association mapping panel (AMP). (XLSX)

S2 Table Number of SNP markers and marker proportion for each chromosome of wheat used for genomewide association mapping. (XLSX)

S3 Table Response to selection based on genomic (GS) and phenotypic and genomic selection (PS+GS) strategies for grain yield validated in different environments for US Pacific Northwest winter wheat. (XLSX)

S4 Table Genomic estimated breeding values (GEBV) for the F5 and DH winter wheat breeding lines under a standard genomic selection (GS1) scenario. (XLSX)

S5 Table Genomic estimated breeding values (GEBV) for the F5 and DH winter wheat breeding lines with GWAS-derived markers included as fixed effects in the prediction model (GS2). (XLSX)

S6 Table Response to selection, *R* based on phenotypic selection (PS) and marker-based selection (MS) for grain yield in US Pacific Northwest winter wheat. *R* calculated relative to the mean of the check lines.
(XLSX)

S7 Table Response to selection, *R* for GEBV-based selection (GS1 and GS2) strategies for grain yield in US Pacific Northwest winter wheat. *R* calculated relative to the mean of the check lines.
(XLSX)

S8 Table Response to selection, *R* for phenotypic + genomic (PS+GS1 and PS+GS2) selection strategies and the number of lines selected in combining both approaches for selection (in parentheses) of yield in US Pacific Northwest winter wheat. *R* calculated relative to the mean of the check lines.
(XLSX)

S9 Table Winter wheat breeding lines selected under a marker-based selection (MS) strategy using the five most significant SNPs identified using a GWAS approach for a diverse winter wheat mapping panel. (XLSX)

S10 Table Roger’s genetic coefficient between the association mapping training panel (AMP) and the winter wheat breeding lines across each chromosome. (XLSX)

S1 File Genotype data (16,233 SNP markers) for the winter wheat association mapping panel (AMP) used for genomewide association study. (XLSX)

S2 File Genotype data (11,089 SNP markers) for the winter wheat breeding lines used for genomic predictions for grain yield. This panel is a subset of the 16,233 markers used for the AMP (S1 File).
(XLSX)

S3 File Adjusted yield (t/ha) for each site-year combination for the winter wheat association mapping panel (AMP). (XLSX)

S4 File Adjusted yield (t/ha) for the F5 and DH winter wheat breeding lines. (XLSX)
