# Supplementary figures and images for: Gains through selection for grain yield in a winter wheat breeding program
Source: PLoS One. 2020 Apr 28;15(4):e0221603. doi: 10.1371/journal.pone.0221603 (PMC7188280; doi:10.1371/journal.pone.0221603)

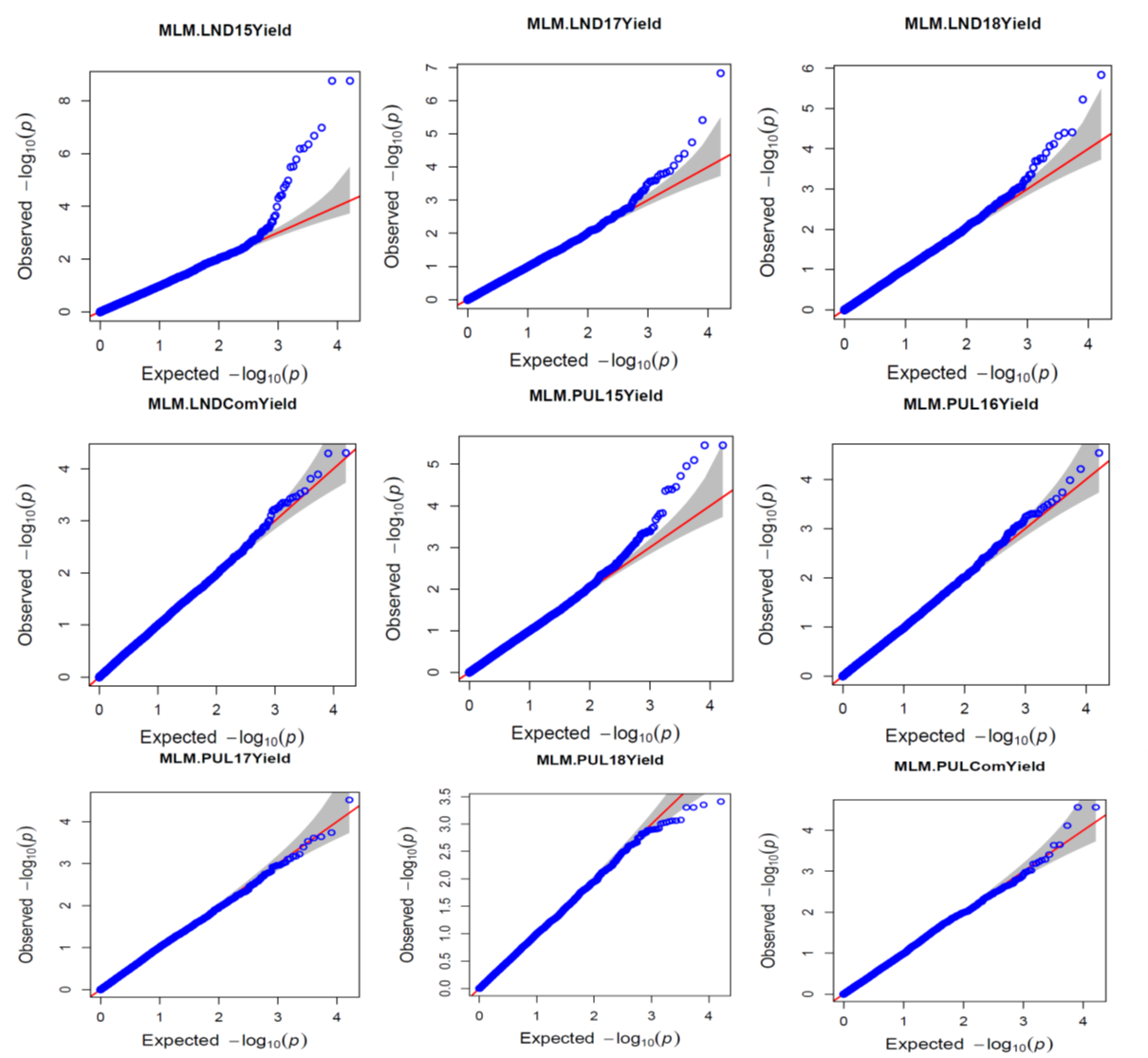

Supplement: S1 Fig — There were minimal deviations from the diagonal line (in red) indicating that the GWAS model (K only) was already able to capture population structure and genetic relatedness, and hence, principal components were excluded in the model. The SNP markers that deviate from the diagonal on the upper right-hand section of the graph are the loci significantly associated with the trait. (TIF) [file pone.0221603.s001.tif]
